# A Novel Silicon Allotrope in the Monoclinic Phase

**DOI:** 10.3390/ma10040441

**Published:** 2017-04-22

**Authors:** Chaogang Bai, Changchun Chai, Qingyang Fan, Yuqian Liu, Yintang Yang

**Affiliations:** Key Laboratory of Ministry of Education for Wide Band-Gap Semiconductor Materials and Devices, School of Microelectronics, Xidian University, Xi’an 710071, China; chaoggangbai@gmail.com (C.B.); ccchai@mail.xidian.edu.cn (C.C.); yuqianliuxd@163.com (Y.L.); ytyang@xidian.edu.cn (Y.Y.)

**Keywords:** silicon allotrope, first-principles calculations, mechanical properties, thermal properties

## Abstract

This paper describes a new silicon allotrope in the *P*2/*m* space group found by first-principles calculations using the Cambridge Serial Total Energy Package (CASTEP) plane-wave code. The examined *P*2/*m*-Si belongs to the monoclinic crystal system. *P*2/*m*-Si is an indirect band-gap semiconductor with a band gap of 1.51 eV, as determined using the HSE06 hybrid functional. The elastic constants, phonon spectra and enthalpy indicate that *P*2/*m*-Si is mechanically, dynamically, and thermodynamically stable. *P*2/*m*-Si is a low-density (2.19 g/cm^3^) silicon allotrope. The value of *B*/*G* is less than 1.75, which indicates that the new allotrope is brittle. It is shown that the difference in the elastic anisotropy along different orientations is greater than that in other phases. Finally, to understand the thermodynamic properties of *P*2/*m*-Si, the thermal expansion coefficient *α*, the Debye temperature *Θ*_D_, and the heat capacities *C*_P_ and *C*_V_ are also investigated in detail.

## 1. Introduction

Silicon plays an extremely important role in semiconductor materials [1,2,3,4,5,6,7,8,9]. In fact, silicon cannot be substituted in the modern semiconductor industry. Almost ninety percent of semiconductor components are based on silicon. In addition, silicon is the second most abundant element on earth, and its processing is most mature at the semiconductor industry level. Moreover, silicon is an optional material in many electronics applications [10]. In particular, silicon is one of the most important photovoltaic (PV) materials in the solar cell industry [2,10]. Monocrystalline, polycrystalline, and amorphous silicon are types of silicon materials used in solar cells. Due to the existing processing conditions, diamond silicon is the primary material used in the solar cell market. However, diamond silicon (space group: *Fd*-3*m*) is not a direct band-gap semiconductor, and there is a large energy difference (2.3 eV) between the indirect band gap and the direct band gap, which reduces its solar energy absorption efficiency [10,11]. Under extreme conditions, a crystal may undergo a phase transition [10], which may reduce the solar energy absorption efficiency. Besides, the band gap of silicone monolayers can be tuned from semimetallic to semiconducting by oxygen adatoms [12]. Thus, researchers continue to search for silicon allotropes with direct band gap or better physical properties.

To meet the needs of industry for materials with efficient solar energy absorption, many silicon allotropes have been reported. Many semiconductor silicon allotropes have been proposed [2,4,8,9,10,11,13]. Wang et al. [4] found six other metastable silicon allotropes with direct or quasi-direct band gaps of 0.39–1.25 eV utilizing Vienna An-initio Simulation Package (VASP) code. They found that these six metastable silicon allotropes not only have a direct band gap or quasi-direct band gap but also have better optical properties than diamond silicon. Recently, Fan et al. [10] investigated four silicon allotropes, including one quasi-direct band-gap phase (*Amm*2) with a band gap of 0.74 eV and three indirect band-gap phases (*C*2/*m*-16, *C*2/*m*-20, and *I*-4) with band gaps of 0.56, 0.53, and 1.28 eV, respectively. The *B*/*G* values indicate that all four phases of silicon are brittle. Moreover, in Ref. [13], Feng et al. not only report the structural properties of two new allotropes, but also elucidate the formation of silicone on an Ag (111) surface. In addition, they found that the two phases have quite similar formation energies and stabilities. Feng et al. [13] obtained silicene by epitaxial growth on conductive substrates. However, the strong silicene-substrate interaction may depress its superior electronic properties [14]. Thus, Du et al. [14] improved the growth method of silicone on Ag (111) by oxygen intercalation and also successfully obtained silicone through the oxidization of bilayer silicene on an Ag (111) surface. In addition, the interactions between the top layer of silicone and the underlying silicene oxide or substrate were weakened by oxidization in this method. Oh et al. [15] presented super-stable pure-silicon superlattice structures that can be applied in solar cell industry and can lead to the realization of pure Si-based optoelectronic devices. Moreover, the minimum band gap of the structure is smaller than that of diamond silicon (the experimental value of diamond silicon 1.12 eV [11]) [15]. While the enthalpy of many of the superlattcies is lower than *P*2/*m* Si. The enthalpy is very close to diamond silicon. In addition, Lee et al. [16] reported two inverse design approaches for the discovery of new photovoltaic materials and successfully predicted favourable structures using the Conformational Space Annealing algorithm. One of the advantages of this approach is that the crystal structures do not need to be known. Kim et al. [17] reported a structure in *Cmcm* space group, and this structure is an indirect band-gap semiconductor with a band gap of 1.41 eV. Besides, they also examined the dynamical stability. And the structure is dynamically stable to 10 GPa. Guo et al. [18] found a missing structure, the h-Si_6_ silicon phase in the *P*6_3_/*mmc* space group, by using silicon triangles as building blocks. Using first-principles calculations, they confirmed that this structure has thermal, dynamical, and mechanical stability. In addition, h-Si_6_ is a direct band-gap semiconductor with a band gap of 0.61 eV and has remarkably better optical properties than diamond silicon. 

In this work, a new silicon allotrope is proposed. The original structure of *P*2/*m*-Si is composed of a lattice similar to that of carbon, in which Si is substituted for C [19]. The calculated formation enthalpy (0.08 eV/atom larger than that of diamond silicon [11]) indicates that *P*2/*m*-Si has a higher thermodynamic stability than that of *tP*16-Si (0.28 eV/atom larger than that of diamond silicon [11]). Moreover, this paper also examines, in detail, physical properties such as structural properties, elastic properties and elastic anisotropic properties. In Ref. [20], the authors present a very similar silicon allotrope with strong absorption in the visible region for photovoltaic applications. In addition, the structure in Ref. [20] has a total energy that is higher than that of diamond silicon by less than 0.15 eV/atom, while the energy of *P*2/*m*-Si is higher than that of diamond silicon by 0.08 eV/atom. This restriction assures that the experimental synthesis of these structures is energetically feasible [20].

## 2. Calculations Methods

In this paper, the Si allotrope in the *P*2/*m* structure is examined using density functional theory (DFT) [21,22] using the Cambridge Serial Total Energy Package (CASTEP) plane-wave code [23]. The Broyden–Fletcher–Goldfarb–Shanno (BFGS) [24] minimization scheme is used to optimize the geometric structure. This work adopts two functionals: the generalized gradient approximation (GGA) [25] and the local density approximation (LDA) [26,27]. First, we obtained the energy cutoff of 340 eV and *k*-point of *P*2/*m*-Si by conducting structural optimizations. The *k*-point grid was 0.025 Å^−1^, which corresponds to 7 × 3 × 8 for *P*2/*m*-Si. Then, the elastic constants were calculated for the structure optimized by the strain-stress method. To investigate the dynamical stabilities of the obtained structures, the phonon spectrum was calculated with different pressures using the linear response approach [28].

## 3. Results and Discussion

**Figure 1:**
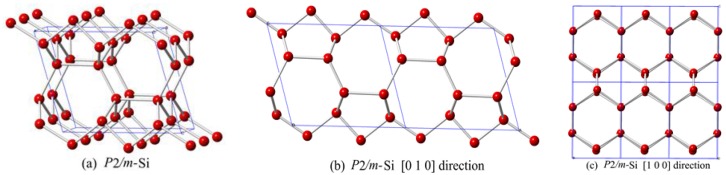
(**a**) Unit cell crystal structures of *P*2/*m*-Si; (**b**) The structural view along the [010] direction; (**c**) The structural view along the [100] direction.

This paper reports a new silicon allotrope in the *P*2/*m* space group. The crystal structure of *P*2/*m-*Si is shown in Figure 1. The structure of *P*2/*m*-Si is composed of six-membered silicon rings that are similar to graphite. The zigzag six-membered silicon ring affords *P*2/*m*-Si a lower density than other silicon allotrope materials. The positions of silicon atoms in *P*2/*m*-Si are Si1 (0.5000, 0.8949, 0.0000), Si2 (0.0000, 0.6741, 0.0000), Si3 (0.0000, 0.3330, 0.0000), and Si4 (0.5000, 0.1340, 0.0000). *P*2/*m*-Si can be described as corrugated graphite sheets interconnected by an alternating sequence of odd 5- or 7-membered rings of silicon, when viewed along the [010] direction, as shown in Figure 1b. However, the structure consists of 6-membered rings of silicon when viewed in the [100] direction. The lattice parameters of *P*2/*m*-Si at zero pressure are shown in Table 1. From Table 1, the calculated lattice parameters of diamond silicon (*a* = 5.426Å) are in excellent agreement with the reported experimental results (*a* = 5.431 Å) [29]. The values obtained by PBE are closer to the experimental values for diamond silicon than the other methods. Thus, the following discussion mainly gives results calculated by the PBE method. In addition, the calculated results in this work for space group *P*222_1_ are consistent with the results of Fan et al. [5].

**Figure 2:**
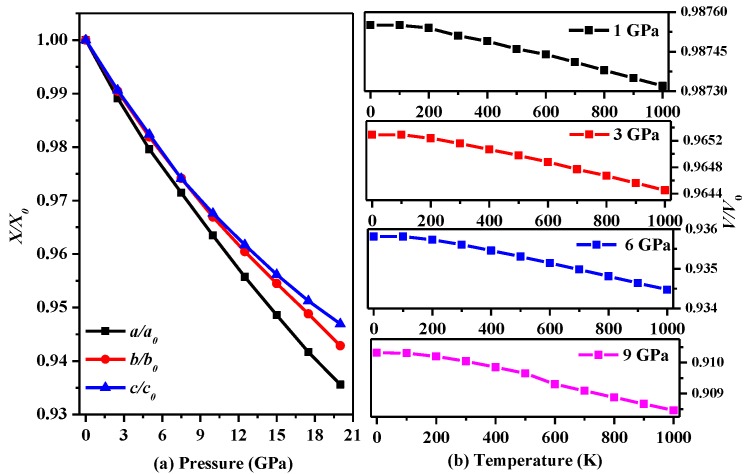
(**a**) The lattice constants *a/a*_0_, *b*/*b*_0_, and *c*/*c*_0_ during compression as functions of pressure for *P*2/*m*-Si; (**b**) The lattice constants *a/a*_0_, *b/b*_0_, and *c/c*_0_ during compression as functions of temperature for *P*2/*m*-Si.

In addition, the influences of temperature and pressure on the lattice parameters are further studied, and the results are shown in Figure 2. Figure 2a presents trends in the lattice constants of *P*2/*m*-Si with increasing pressure. As seen in Figure 2a, it is very difficult to compress *P*2/*m*-Si in the *c*-axis direction, while compression in the *a*-axis direction is the easiest. In addition, the difference in the lattice parameters of *P*2/*m*-Si and diamond silicon along the *a*-axis is larger than that along the other axes. In Ref. [5], *P*222_1_-Si is shown to be compressed easily in the *b*-axis direction. In addition, the decline in the slope of the lattice parameters lessens when the pressure is larger than 12 GPa. Figure 2b describes the trend in the volume with increasing pressure at different temperatures. The trend in the volume at different temperatures remains roughly the same as the pressure increases, indicating that this material may be used in environments that undergo serious temperature changes.

The elastic constants C_ij_, bulk modulus *B*, shear modulus *G* and Young’s modulus *E* of *P*2/*m*-Si calculated by the PBE method are given in Table 2. Table 2 also lists the elastic constants and the elastic modulus of diamond silicon and other silicon allotropes. Monoclinic symmetry gives 13 independent elastic constants [32], which are listed in Table 2. If the allotrope is a stable monoclinic structure, the elastic constants of *P*2/*m*-Si should satisfy the mechanical stability criteria of monoclinic symmetry [32].

(1) $$C_{\mathrm{ii}}>0,i=1...6$$

(2) $$[C_{11}+C_{22}+C_{33}+2(C_{12}+C_{13}+C_{23})]>0$$

(3) $$(C_{33}C_{55}-C_{35}^{2})>0$$

(4) $$(C_{44}C_{66}-C_{46}^{2})>0$$

(5) $$(C_{22}+C_{33}-2C_{23})>0$$

(6) $$[C_{22}(C_{33}C_{55}-C_{35}^{2})+2C_{23}C_{25}C_{35}-C_{23}^{2}C_{55}-C_{25}^{2}C_{33}]>0$$

(7) $$g=C_{11}C_{22}C_{33}-C_{11}C_{23}^{2}-C_{22}C_{13}^{2}-C_{33}C_{12}^{2}+2C_{12}C_{13}C_{23}$$

(8) $$\begin{array}{l} 2[C_{15}C_{25}(C_{33}C_{12}-C_{13}C_{23})+C_{15}C_{35}(C_{22}C_{13}-C_{12}C_{23})\\ +C_{25}C_{35}(C_{11}C_{23}-C_{12}C_{13})]-[C_{15}^{2}(C_{22}C_{33}-C_{23}^{2})+C_{25}^{2}(C_{11}C_{33}-C_{13}^{2})\\ +C_{35}^{2}(C_{11}C_{22}-C_{12}^{2})]+C_{55}g>0 \end{array}$$

**Figure 3:**
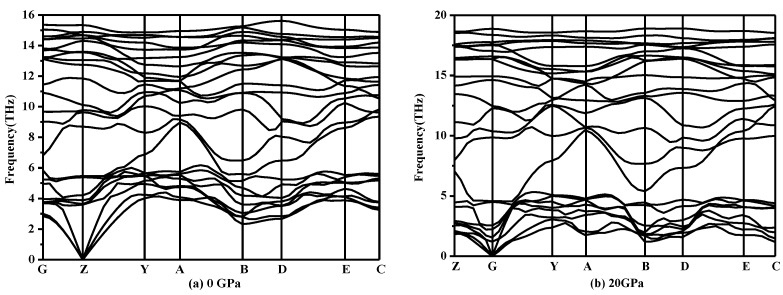
Phonon spectra for *P*2/*m*-Si at different pressures: (**a**) 0 GPa and (**b**) 20 GPa.

From Table 2, as the mechanical stability criteria of monoclinic symmetry are satisfied, it can be concluded that *P*2/*m*-Si is mechanically stable [34]. Meanwhile, the phonon spectra of *P*2/*m*-Si was also investigated under different pressures. The phonon spectra should show no imaginary frequencies over the entire Brillouin zone, which indicates that the phase is dynamically stable [35]. The phonon spectra of *P*2/*m*-Si at different pressures are shown in Figure 3. The phonon spectra of *P*2/*m*-Si shows no imaginary frequencies over the entire Brillouin zone between 0 GPa and 20 GPa, which indicates that *P*2/*m*-Si is dynamically stable up to 20 GPa. Above this pressure, *P*2/*m*-Si is destabilized, indicating that a structural transformation occurs.

**Figure 4:**
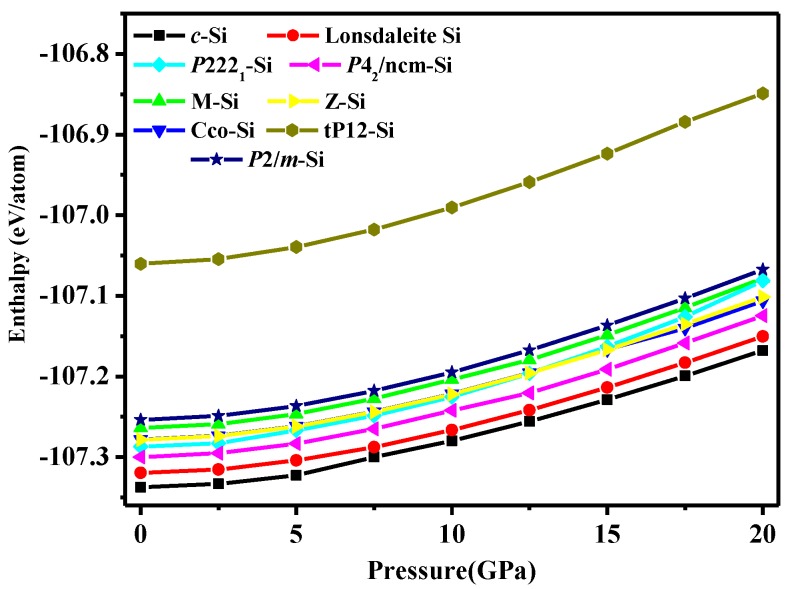
The calculated enthalpies of different silicon structures as a function of pressure.

The enthalpy of *P*2/*m*-Si is an important factor in the stability, which measures the thermodynamic stability. The enthalpies of different silicon allotropes as the pressure increases are shown in Figure 4. It is clear that diamond silicon is the most stable phase over the entire pressure range in Figure 4. In Ref. [11] and this work, the most unfavourable structure is the *tP*12 phase (which is higher in energy than diamond silicon by 0.276 and 0.269 eV/atom at zero pressure in this work and in Ref. [4], respectively), but *tP*12-Si is still dynamically and mechanically stable [4]. The curve of *tP*12-Si is also shown in Figure 4, while the energy of *P*2/*m*-Si is lower than that of *tP*12-Si by 0.19 eV/atom at zero pressure and 0.22 eV/atom at a pressure of 20 GPa. Simultaneously, *P*2/*m*-Si is higher in energy than diamond silicon by 0.08 eV/atom at zero pressure. In addition, the enthalpy of *P*2/*m*-Si is smaller than that of *Amm*2 (−170.23 eV/atom), *C*2/*m*-16 (−170.21 eV/atom), *C*2/*m*-20 (−170.24 eV/atom), and *I*-4 Si (−170.24 eV/atom) [10], respectively. The calculated formation enthalpy indicates that *P*2/*m*-Si has a higher thermodynamic stability than that of *Amm*2 Si, *C*2/*m*-16 Si, *C*2/*m*-20 Si, *I*-4 Si, and superlattices. There are many intermediate phases between *P*2/*m*-Si and diamond silicon, and the excess enthalpy of each structure relative to diamond silicon is shown in Table 3. Most of the structures have enthalpies higher than diamond silicon (−170.34 eV/atom). Although the energies of *M*-Si, *Cco*-Si, *P*222_1_-Si, *Z*-Si, *tP*12-Si, and *P*2/*m*-Si are higher than that of diamond silicon, while their enthalpies are very close to that of diamond silicon. The order of enthalpy from high to low is *tP*12-Si > *P*2/*m* Si > *M* Si > *Pmmn* Si > *Cco* Si > *Pbam* Si > *P*222_1_ Si > *P*4_2_/*ncm* Si > *Cmcm* Si > *P*2_1_/*m* Si > Lonsdaleite Si. The enthalpy of many of the superlattcies is lower than *P*2/*m* Si. However, *tP*12-Si is still thermodynamically dynamically and mechanically stable. So the *P*2/*m* Si is thermodynamically stable.

Table 2 lists the bulk moduli, shear moduli, Young’s moduli, and Poisson’s ratios of *P*2/*m*-Si, diamond silicon and other silicon allotropes. The bulk modulus and shear modulus are both important characteristics of a material. The bulk modulus and shear modulus represent the resistance to fracture and plastic deformation, respectively [34]. The bulk modulus of *P*2/*m*-Si is slightly smaller than that of diamond silicon. The shear modulus of *P*2/*m*-Si is also slightly smaller than that of diamond silicon but is the greatest among those of *P*2/*m*-Si, *C*2/*m*-16 Si, *C*2/*m*-20 Si, and *I*-4 Si [10]. The hardness of a material can determine the plastic and elastic properties to some extent and can be predicted by following formula [34]:

(9) $$H_{V}=2{{(k}^{2}G)}^{0.585}-3$$

In the above formula, *k* is equal to *G*/*B*. The calculated hardness of *P*2/*m*-Si is 10.0 GPa, while the hardness of diamond silicon is 12.4 GPa. However, the hardness of *P*2/*m*-Si is slightly larger than that of the *C*2/*m*-16, *C*2/*m*-20, *I*-4 and *Amm*2 phases (*C*2/*m*-16: 8.4 GPa, *C*2/*m*-20: 9.8 GPa, *I*-4: 7.6 GPa, *Amm*2: 9.1 GPa) [10]. In *P*2/*m*-Si, there are eight different bond lengths: 2.3945, 2.4582, 2.3365, 2.3420, 2.3274, 2.3522, 2.3008, and 2.4099 Å, and the average bond length is 2.3652 Å, which is slightly smaller than that of diamond silicon (2.3729 Å) [10]. The bond lengths of *P*2/*m*-Si are very close to that of diamond silicon, indicating that the bond energy of the Si-Si bonds should be as high as that of diamond silicon. *P*2/*m*-Si has the smallest average bond length among the *C*2/*m*-16, *C*2/*m*-20, *I*-4, and *Amm*2 phases, and thus the hardness of *P*2/*m*-Si is the greatest. On the other hand, there is only one bond length in diamond silicon, but in the other silicon allotropes, including *P*2/*m*-Si, there are seven, eight, and even nine or more bond lengths in which the extra bond lengths are all larger than that of diamond silicon. Thus, the bond energy of these extra Si-Si bonds is weaker than that of diamond silicon, making the hardness of the other silicon allotropes, including *P*2/*m*-Si, lower than that of diamond silicon. 

The *B*/*G* ratio of *P*2/*m*-Si is 1.48, as calculated by the PBE method, meaning *P*2/*m*-Si is a brittle material, according to the theory proposed by Puge in which the *B*/*G* value of a brittle material is less than 1.75 [36,37]. The *B*/*G* ratio of *P*2/*m*-Si is slightly greater than that of diamond silicon (1.38); that is to say, diamond silicon is more brittle. The *B*/*G* ratio of *C*2/*m*-16 Si is 1.60, *C*2/*m*-20 Si is 1.50, *I*-4 Si is 1.68, *Amm*2 Si is 1.54 [10], and *P*222_1_ Si is 1.54 [5]. Therefore, *P*2/*m*-Si is the most brittle among *C*2/*m*-16 Si, *C*2/*m*-20 Si, *I*-4 Si, *Amm*2 Si, and *P*222_1_ Si. In addition, Poisson’s ratio *v*, which gives the ratio of the fraction of expansion and the fraction of compression, can be obtained by the following equation [26]:

(10) $$v=\frac{3B-2G}{2(3B+G)}$$

According to Ref. [38], the Poisson’s ratio *v* of a ductile material is usually larger than 0.26, while brittle materials usually have a small *v* (*v* < 0.26) [38]. Moreover, the Poisson’s ratio *v* of *P*2/*m*-Si is consistent with its *B*/*G*, and *P*2/*m*-Si is characterized as a brittle material via its Poisson’s ratio *v*. At the same time, the Poisson’s ratio of *Amm*2 (0.23) is slightly larger than that of *P*2/*m*-Si. The Poisson’s ratio of *I*-4 Si is the largest (0.25), and that of diamond silicon is the smallest (0.21) by only a difference of 0.04 [10]. 

Young’s modulus, which is calculated from the bulk modulus and shear modulus in Formula (11) [39], is the physical attribute describing the resistance of the material to deformation.

(11) $$E=\frac{9BG}{3B+G}$$

According to the listed values of *E* in Table 2, the Young’s modulus of *P*2/*m*-Si is smaller than that of diamond silicon and larger than that of *C*2/*m*-20, *I*-4, *Amm*2 and *C*2/*m*-16 Si. That is, the mechanical properties of *P*2/*m*-Si are the greatest among *C*2/*m*-20, *I*-4, *Amm*2 and *C*2/*m*-16 Si.

It is well known that the elastic anisotropy is an important parameter in engineering science and crystal physics. Crystals show different physical and chemical properties in different directions. In addition, the anisotropy represents the extent of this property. The universal anisotropic index *A*^U^ and percentage of elastic anisotropy for the bulk modulus and shear modulus can be calculated by the following equations [39,40]:

(12) $$A_{B}=\frac{B_{V}-B_{R}}{B_{V}+B_{R}}$$

(13) $$A_{G}=\frac{G_{V}-G_{R}}{G_{V}+G_{R}}$$

(14) $$A^{U}=5\frac{G_{V}}{G_{R}}+\frac{B_{V}}{B_{R}}-6$$

where *B*_V_ represents the bulk modulus found using the Voigt method and *B*_R_ represents the bulk modulus found using the Reuss method. In addition, *G*_V_ and *G*_R_ represent the shear modulus found using the Voigt and Reuss method, respectively. *A*^U^ takes both bulk and shear effects into consideration, and the extent of anisotropy increases with larger values of *A*^U^ [4,26,41]. The interrelated factors of elastic anisotropy for *P*2/*m*-Si are shown in Table 4. From Table 4, the *A*^U^ of *P*2/*m*-Si is smaller than that of diamond silicon. The order from large to small according to *A*^U^ in Table 4 is diamond silicon > *P*2/*m* Si > *C*2/*m*-20 Si > *C*2/*m*-16 Si > *Amm*2 Si > *I*-4 Si [10]. *A*_B_ and *A*_G_ of the *P*2/*m* phase are the smallest among the listed allotropes, which indicates that it has least anisotropy in its shear modulus among these silicon allotropes [34]. The *A*_B_ values of *P*2/*m*-Si and *I*-4 Si are almost the same as that of diamond silicon, which indicates that they have the least anisotropy in the bulk modulus, similar to diamond silicon.

The shear anisotropy factors are calculated in this work to verify the *P*2/*m*-Si elastic anisotropy along different orientations (shown in Table 4). In addition, Table 4 also lists the compression anisotropy factors and the shear anisotropy factors of *P*2/*m*-Si and the *C*2/*m*-20, *C*2/*m*-16, *Amm*2, *I*-4, and *Fd*-3*m* phases. The value of the shear anisotropy factors represents the level of anisotropy in the bonding between atoms in different planes. The three formulas defining the shear anisotropy factors are as follows [34,42]:

(15) $$A_{1}=\frac{4C_{44}}{C_{11}+C_{33}-2C_{13}}$$

(16) $$A_{2}=\frac{4C_{55}}{C_{22}+C_{33}-2C_{23}}$$

(17) $$A_{3}=\frac{4C_{66}}{C_{11}+C_{22}-2C_{12}}$$

where *A*_1_ is the factor for the (100) shear plane between the [011] and [010] directions, *A*_2_ is for the (010) shear plane between the [101] and [001] directions, and *A*_3_ is for the (001) shear plane between the [110] and [010] directions. A value with a larger offset than 1 implies that the crystal behaves more like an anisotropic material, and a value in the vicinity of 1 indicates an isotropic crystal. The results in Table 4 indicate that *P*2/*m*-Si shows more distinct performance. The shear anisotropy factors of *P*2/*m*-Si along different directions are very different. In particular, there is a larger offset between *A*_3_ and 1, while the other two directions are close to 1. The value for the three directions of diamond silicon are the all smaller than 1 by 0.227 (diamond silicon: *A*_1_: 0.773). It is clear that *P*2/*m*-Si exhibits a larger anisotropy in *A*_3_ than that of diamond silicon, while *P*2/*m*-Si exhibits a smaller anisotropy in *A*_1_ and *A*_2_ than diamond silicon. Meanwhile, the *Amm*2 and *C*2/*m*-20 phases exhibit a larger anisotropy in *A*_3_ than diamond silicon, while the *I*-4 phase exhibits the smallest anisotropy in *A*_1_ and *A*_2_.

**Figure 5:**
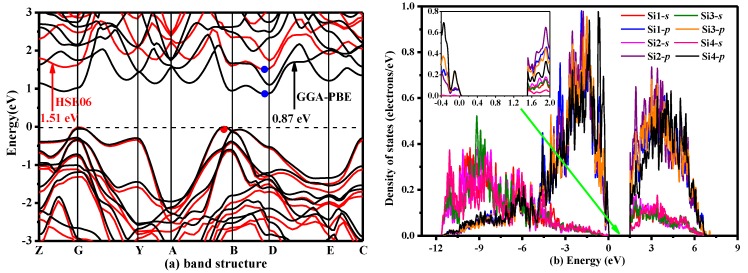
(**a**) Electronic band structure and (**b**) density of states of *P*2/*m*-Si.

The fundamental physical and chemical properties of materials depend on the electronic structure. However, the DFT method typically underestimates the electronic band structure [36]. The true band gap is usually larger than the simulation result. The Heyd-Scuseria-Ernzerhof (HSE06) functional was examined in consideration of this problem. The results of HSE06 are relatively accurate. The hybrid functional HSE06 was used in the following form [36,43,44]:

(18) $$E_{xc}^{\mathrm{HSE}}=\mu E_{x}^{\mathrm{HF},\mathrm{SR}}(\omega )+(1-\mu )E_{x}^{\mathrm{PW}91,\mathrm{SR}}(\omega )+E_{x}^{\mathrm{PW}91,\mathrm{LR}}(\omega )+E_{c}^{\mathrm{PW}91}$$

where the HF mixing parameter *μ* is 0.25 and the screening parameter *ω* is 0.207 Å^−1^ [44,45]. The electronic band structure of *P*2/*m*-Si was calculated using the GGA-PBE and HSE06 functionals, and the electronic band structures of *P*2/*m*-Si are shown in Figure 5a. Figure 5a clearly shows that *P*2/*m*-Si is an indirect-band-gap semiconductor with a band gap of 1.51 eV, as determined by the HSE06 hybrid functional. The result calculated by the HSE06 hybrid functional is much larger than the result from the GGA-PBE functional (0.87 eV). In addition, the band gap was found to be higher than that of diamond silicon by 0.23 eV using the same calculation method (diamond silicon: 1.28 eV [11,44]). Moreover, the band gap of diamond silicon calculated by HSE06 is very close to the experimental value (1.12 eV [11,30]). The charge effective mass, which is also a key physical property for applications, can be obtained by the formula m^*^ = *h*^2^/(*d*^2^*E*/*d*^2^*k*) in which *h* is the planck constant, *E* is energy, *k* is wave vector. (m^*^_n_ = 0.023m_0_, m^*^_p_ = 0.019m_0_.). The effective mass reflects the carrier mobility indirectly (*μ* = *qτ*/m^∗^). The experimental effective mass at the *Γ* valley for *a-*GaN is 0.2m_0_ [46], while the effective mass for a hole in *a*-GaN at the valence band is 0.6m_0_. Besides, the effective mass at the *Γ* valley for GaAs is 0.0635m_0_ at the temperature 300 K [47]. The effective mass at the *Γ* valley for Ge is 0.038m_0_ at the temperature 300 K. The averaged light-hole mass in [001] is 0.046m_0_ [48]. The calculated effective mass of *P*2*/m*-Si is much smaller than that of GaN. Therefore, the mobility of *P*2/*m*-Si is larger than that of GaN, because the mobility is inversely proportional to the effective mass (μ = qτ/m^∗^). The electronic mobility of *P*2/*m*-Si is close to Ge. The density of states of *P*2/*m*-Si is displayed in Figure 5b, and the inset figure illustrates the local features at the Fermi level in detail. The dashed line represents the Fermi level. The DOS is divided into three regions: −13.5 eV to −5 eV, −5 eV to 0 eV and 0 eV to 7 eV. The lowest band ranging from −13.5 eV to −5 eV is characterized by the Si1-*s*, Si2-*s*, Si3-*s* and Si4-*s* orbitals. The middle band ranging from −5 eV to 0 eV is characterized by the Si1-*p*, Si2-*p*, Si3-*p* and Si4-*p* orbitals, and the upper band is also characterized by the Si1-*p*, Si2-*p*, Si3-*p* and Si4-*p* orbitals. In addition, the energy of the upper band is above 0.86 eV.

**Figure 6:**
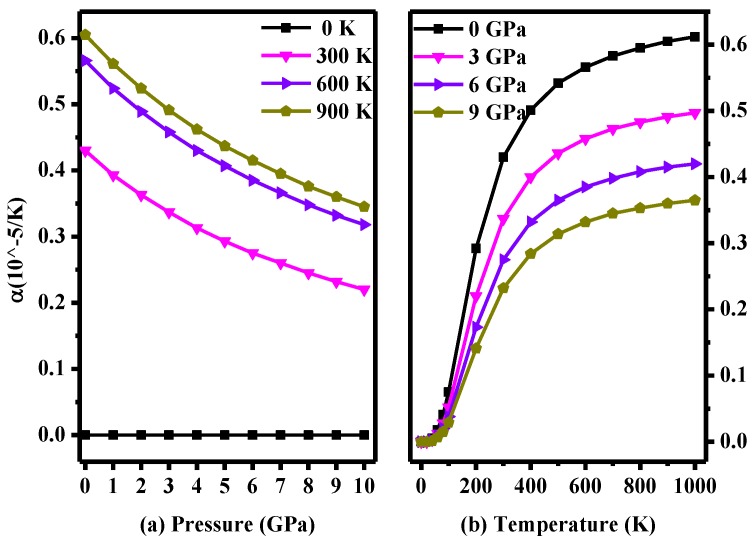
(**a**) Pressure dependence of the thermal expansion coefficient for *P*2/*m*-Si; (**b**) Temperature dependence of the thermal expansion coefficient for *P*2/*m*-Si.

The thermodynamic properties of semiconductor materials at higher temperatures and pressures are interesting [38]. In this work, the highest pressure examined is 10 GPa, and the highest temperature is 1000 K. In the above conditions, the thermal expansion coefficient *α*, Grüneisen parameter *γ* and heat capacity were calculated. Figure 6 shows the thermal expansion coefficient *α* for *P*2/*m*-Si as functions of temperature and pressure. Figure 6a presents the relationship between the thermal expansion coefficient and pressure at different temperatures. The reduction in the amplitude decreases as the pressure increases, as shown in Figure 6a, and the difference in *α* is small at the same temperature. The reduction in amplitude at lower pressure is larger than that at higher pressure. However, the thermal expansion coefficient is constant when the temperature is 0 K (*α* = 0 × 10^−5^/K), and the difference in *α* between 600 K and 900 K (0.04 × 10^−5^/K at 0 GPa) is smaller than that between 300 K and 600 K (0.24 × 10^−5^/K at 0 GPa). As seen in Figure 6b, the thermal expansion coefficient increases quickly with increasing temperature, especially when the temperature is below 500 K. The increase in amplitude becomes very small when the temperature is over 500 K. Moreover, the thermal expansion coefficient increases less at increased pressure. Thus, by comparing Figure 6a with Figure 6b, it can be determine that the expansion coefficient *α* is more sensitive to the temperature when the temperature is below 500 K. From Figure 6, it is clear that the effect of pressure on the expansion coefficient *α* is not as significant as the effect of temperature over calculated pressure and temperature ranges.

**Figure 7:**
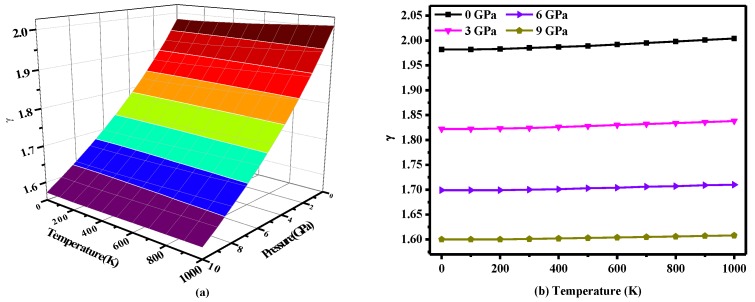
(**a**) Three-dimensional contour plot of the Grüneisen parameter versus pressure and temperature for *P*2/*m*-Si; (**b**) Temperature dependence of the Grüneisen parameter for *P*2/*m*-Si.

Figure 7 shows the change in the Grüneisen parameter *γ* with temperature and pressure, where the Grüneisen parameter *γ* is expressed as *γ = −(dlnΘ(V)*/*dlnV)* [49], where *Θ* is the Debye temperature and *V* is the volume. This parameter describes the anharmonic effects in the lattice vibrations [38]. As seen in Figure 7a, the *γ* parameter reduces by 0.045 per GPa, and the values are very close at different temperatures. In other words, the effect of pressure on the Grüneisen parameter *γ* is greater than that of temperature. As seen in Figure 7b, the *γ* parameter is not very sensitive to temperature. As the temperature increases to 1000 K, the Grüneisen parameter *γ* only increases by 0.02 at 6 GPa and 0.01 at 7 GPa or more.

**Figure 8:**
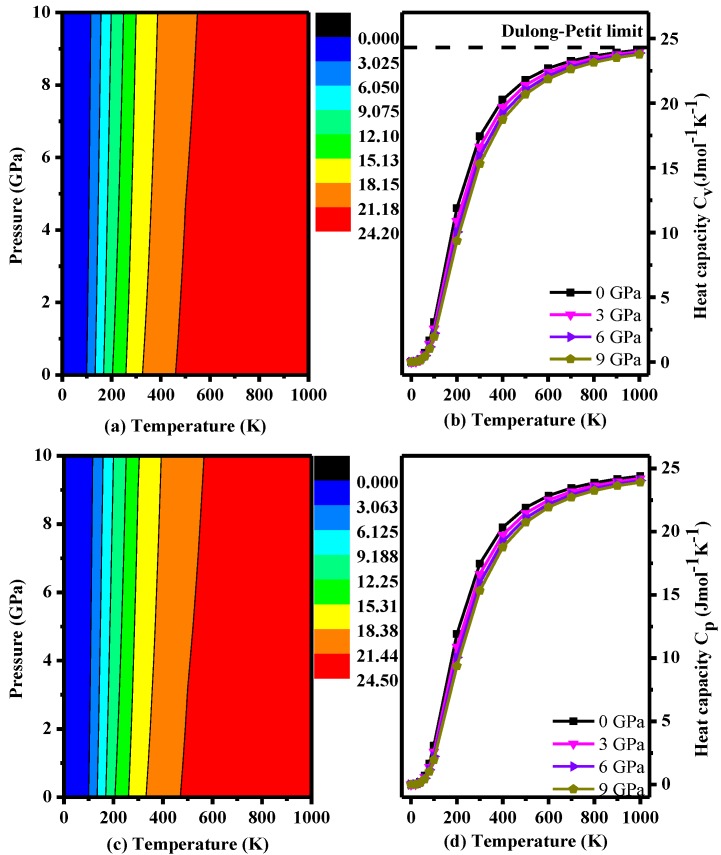
Calculated specific volume *C*_V_ and pressure heat capacity *C*_P_ as a function of pressure for *P*2/*m*-Si at different temperatures: (**a**) *C*_V_ contours; (**b**) *C*_V_–T; (**c**) *C*_P_ contours; and (**d**) *C*_P_–T.

This study also examined the heat capacity at constant volume (*C*_V_) and constant pressure (*C*_P_) (Figure 8), which were calculated by the following formulas [38]:

(19) $$C_{V}=3nk\left[4D\left(\frac{\Theta }{T}\right)-\frac{3\Theta /T}{e^{\Theta /T}-1}\right]$$

(20) $$C_{P}=C_{V}(1+\alpha \gamma T)$$

where *n* is the number of atoms per formula unit, *Θ* is the Debye temperature, *k* is Boltzmann constant and D (*Θ/T*) represents the Debye integral [38]. As seen in Figure 8, the difference between *C*_P_ and *C*_V_ is very small. In addition, the trend is basically consistent with the trend at different pressures. Both plots increase as the temperature increases. It is obvious that the heat capacity is sensitive to temperature when *T* < 500 K; however, the magnitude of the increasement becomes very small when the temperature is over 500 K. In addition, the effect of pressure on the heat capacity is very small. *C*_V_ stays close to constant at the Dulong-Petit limit (24.92 J·mol^−1^·K^−1^) [38] at high temperature and high pressure. Thus, the heat capacity of *P*2/*m*-Si is mainly affected by the temperature, and the effect of the pressure is small compared to that of the temperature.

## 4. Conclusions

This study predicts a new silicon allotrope with space group *P*2/*m*. The crystal structure, mechanical properties, elastic anisotropy, stability, electronic properties, and thermal properties of *P*2/*m*-Si were systematically studied. *P*2/*m*-Si is an indirect-band-gap semiconductor with a band gap of 1.51 eV, and Si in the *P*2/*m* phase is also mechanically, dynamically and thermally stabile at ambient pressures. The hardness was calculated to be 10.0 GPa, which is very close to that of diamond silicon. In addition, *P*2/*m*-Si is more brittle than the other allotropes of *C*2/*m*-16 Si, *C*2/*m*-20 Si, *I*-4 Si, and *Amm*2 Si. *P*2/*m*-Si also has larger anisotropy factors in different planes, especially for *A*_3_. *P*2/*m*-Si has the least anisotropy in its shear modulus, which is similar to diamond silicon, and the universal anisotropy of *P*2/*m*-Si is smaller than that of diamond silicon. High pressure leads to a smaller thermal expansion and a smaller Grüneisen parameter at ambient temperatures. Moreover, the lattice constants, Grüneisen parameter and the heat capacity have very small changes at different temperatures with increasing pressure. In addition, the heat capacity is nearly stable, showing value of 25 J·mol^−1^·K^−1^ when the temperature is higher than 800 K.

## Figures and Tables

**Table 1:** The lattice parameters (Å) of different space groups calculated by different methods.

Method	PBE				PBEsol				CA-PZ			
Space Group	*a*	*b*	*c*	*β*	*a*	*b*	*c*	*β*	*a*	*b*	*c*	*β*
*P*2/*m*	7.253	3.868	6.294	105.9	7.243	3.861	6.283	105.8	7.136	3.802	6.187	105.8
*P*222_1_	5.429	13.112	5.265	-	5.334	12.98	5.211	-	5.349	12.758	5.264	-
*P*222_1_^c^	5.448	13.017	5.339	-	5.445	12.995	5.327	-	5.364	12.803	5.248	-
*P*222_1_	7.495	5.359	5.410	-	7.477	5.432	5.316	-	7.345	5.379	5.258	-
*P*222_1_^f^	7.487	5.450	5.221	-	7.487	5.446	5.206	-	7.365	5.373	5.123	-
*Fd-3m*	*a* = 5.426, 5.402 *^a^*, 5.429 *^e^*, 5.465 *^b^*, 5.431 *^d^*				*a* = 5.466				*a* = 5.374, 5.392 ^a^			

^a^ Ref. [1]; ^b^ Ref. [3]; ^c^ Ref. [5]; ^d^ Ref. [29]-experimental. ^e^ Ref. [30]; ^f^ Ref. [31];

**Table 2:** The calculated elastic constants *C*_ij_, bulk modulus *B*, shear modulus *G*, Young’s modulus *E*, and Poisson’s ratio *v* of different silicon allotropes (GPa) (S.G.: space group).

*S.G.*	Method	*C*_11_	*C*_22_	*C*_33_	*C*_44_	*C*_55_	*C*_66_	*C*_12_	*C*_13_	*C*_15_	*C*_23_	*C*_25_	*C*_35_	*C*_46_	*B*	*G*	*B/G*	*E*	*v*
*P2/m*	GGA	159	167	150	63	55	40	29	42	−1	51	9	2	8	80	54	1.48	132	0.22
	LDA	171	176	160	61	57	40	35	50	0	58	10	3	8	88	54	1.63	134	0.25
*C2/m-16 ^a^*	GGA	146	146	164	48	53	53	51	47	-	43	-	-	-	82	51	1.61	127	0.24
*C2/m-20 ^a^*	GGA	184	167	143	55	52	52	36	46	-	46	-	-	-	83	55	1.51	135	0.23
*I-4 ^a^*	GGA	142	142	145	57	47	55	48	50	-	50	-	-	-	80	48	1.68	120	0.25
*Amm2 ^a^*	GGA	161	179	131	44	44	51	37	42	-	38	-	-	-	78	51	1.54	126	0.23
*Fd-3m*	GGA	154	-	-	79	-	-	56	-	-	-	-	-	-	88	64	1.38	155	0.21
-	LDA	163	-	-	80	-	-	58	-	-	-	-	-	-	93	68	1.37	164	0.21
-	Exp. ^b^	166	-	-	80	-	-	64	-	-	-	-	-	-	102	-	-	-	-

^a^ Ref. [10]; ^b^ Ref. [33].

**Table 3:** The excess formation enthalpy of different silicon allotropes relative to diamond silicon under 0 GPa. (eV). And n represents the Si (111)*n*/Si(SC) superlattices with the cubic- and hexagonal-stacking sequences of Si (111) layers (SM: simple monoclinic, SO: simple orthorhombic, and BCO: base-centered orthorhombic).

**Diamond Si ^a^**	***Lonsdaleite ^a^***	***M*^a^**	***Cco*^a^**	***P*222_1_^a^**	***P*4_2_/*ncm*^a^**	***Z*^a^**	***tP*12 ^a^**	***P*2/m**	***P*2_1_/m ^b^**
0.00	0.02	0.08	0.06	0.05	0.04	0.06	0.28	0.09	0.02
***Imma*^b^**	***Pbam*^b^**	***Pmmn*^b^**	***Cmcm*^b^**	***P*-1 ^c^**	***P*2_1_/c ^c^**	***C*2/*m*-16 ^a^**	***C*2/*m*-20 ^a^**	***I*-4 ^a^**	***Amm*2 ^a^**
0.07	0.06	0.08	0.04	0.13	0.14	0.13	0.10	0.10	0.11
**n = 1 (BCO) ^d^**	**n = 2 (SM) ^d^**	**n = 3 (SM) ^d^**	**n = 4 (SM) ^d^**	**n = 5 (SM) ^d^**	**n = 6 (SM) ^d^**	**n = 7 (SM) ^d^**	**n = 8 (SM) ^d^**	**n = 9 (SM) ^d^**	**n = 10 (SM) ^d^**
0.09	0.05	0.04	0.03	0.03	0.02	0.02	0.02	0.02	0.01
**n = 2 (SO) ^d^**	**n = 3 (SM) ^d^**	**n = 4 (SO) ^d^**	**n = 5 (SM) ^d^**	-	-	-	-	-	-
0.07	0.05	0.03	0.03	-	-	-	-	-	-

^a^ Ref. [10]; ^b^ Ref. [35]; ^c^ Ref. [20]; ^d^ Ref. [15].

**Table 4:** The compression and shear anisotropy percent factors (*A*_B_ and *A*_G_), universal anisotropy indexes (*A*^U^) and shear anisotropy factors (*A*_1_, *A*_2_ and *A*_3_) of different structures.

Space Group	*A*_B_	*A*_G_	*A*^U^	*A*_1_	*A*_2_	*A*_3_
*P2/m*	0.000	0.028	0.269	1.056	1.036	0.578
*Amm*2 *^a^*	0.641	1.359	0.151	0.839	0.750	0.766
*C2/m*-16 *^a^*	0.128	1.475	0.152	0.881	0.940	1.111
*C2/m*-20 *^a^*	0.347	1.971	0.208	0.937	0.954	0.752
*I*-4 ^a^	0.014	0.691	0.070	1.006	1.006	1.167
*Fd*-3*m* *^a^*	0.000	3.500	0.336	0.773	0.773	0.773

^a^ Ref. [10].
