# Correction: A Novel Silicon Allotrope in the Monoclinic Phase. *Materials* 2017, *10*, 441

**DOI:** 10.3390/ma10050561

**Published:** 2017-05-22

**Authors:** Chaogang Bai, Changchun Chai, Qingyang Fan, Yuqian Liu, Yintang Yang

**Affiliations:** Key Laboratory of Ministry of Education for Wide Band-Gap Semiconductor Materials and Devices, School of Microelectronics, Xidian University, Xi’an 710071, China; chaoggangbai@gmail.com (C.B.); ccchai@mail.xidian.edu.cn (C.C.); yuqianliuxd@163.com (Y.L.); ytyang@xidian.edu.cn (Y.Y.)

In the published manuscript “A Novel Silicon Allotrope in the Monoclinic Phase. *Materials* 2017, 10, 441” [1], we wrongly listed the coordinates of the new silicon allotrope. The correct coordinates are Si1 (0.1114, 0.5000, 0.8949), Si2 (0.1217, 0.0000, 0.6741), Si3 (0.5411, 0.0000, 0.3330), and Si4 (0.4194, 0.5000, 0.1340). We emphasize that all our results obtained are reproducible using the four values quoted above and apologize for any inconvenience this has caused.

We thank Prof. Davide M. Proserpio for finding this error.

The changes do not affect the results. The manuscript will be updated and the original will remain available on the article webpage.
